# Sensitive ratiometric sensor for Al(III) detection in water samples using luminescence or eye-vision

**DOI:** 10.1007/s44211-023-00340-6

**Published:** 2023-04-18

**Authors:** Gasser M. Khairy, Alaa S. Amin, Sayed M. N. Moalla, Ayman Medhat, Nader Hassan

**Affiliations:** 1 Chemistry Department, Faculty of Science, Suez Canal University, Ismailia, 41522 Egypt; 2 Chemistry Department, Faculty of Science, Benha University, Benha, 13518 Egypt; 3 Chemistry Department, Faculty of Science, Port Said University, Port Said, 42526 Egypt

**Keywords:** Al(III) determination, Lanthanide complexes, Ratiometric, Drinking water, Optical sensor, Environmental analysis

## Abstract

A facile, quick, and sensitive ratiometric luminescence sensor is designed for detection aluminum ions in water samples using luminescence or eye-vision. This approach relies on the emission change of the europium(III) complex with 3-(2-naphthoyl)-1,1,1,-trifluoro acetone (3-NTA) after interaction with various concentration of aluminum ions. The addition of aluminum ions suppressed the Eu(III) emission at 615 nm under 333 nm excitation, while simultaneously enhancing the ligand emission at 480 nm. Optimum detection was obtained in methanol. The quantification of aluminum ions using ratiometric method was determined by plotting the luminescence ratio (F_480nm_/F_615nm_) *versus* aluminum ions concentration. The calibration plot was obtained within the range 0.1–100 µM with LOD = 0.27 µM. Additionally, the concentration of aluminum ions can be estimated semi-quantitatively by visually observing the luminescence colour change of the probe from red to light green and then to dark green after being excited by a UV lamp with 365 nm. As far as we are aware, this is the first luminescent lanthanide complex-based ratiometric probe for the detection of aluminum ions. The probe showed remarkable aluminum ions selectivity relative to that of other metal ions. The suggested sensor was used effectively to identify aluminum ions in water samples with good results.

**Graphical Abstract:**

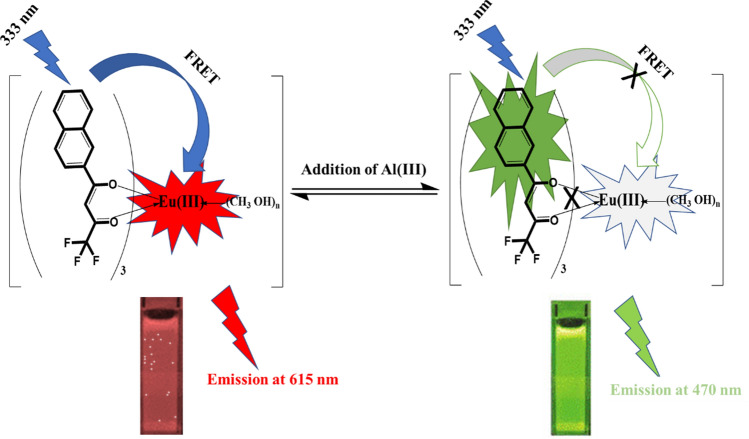

**Supplementary Information:**

The online version contains supplementary material available at 10.1007/s44211-023-00340-6.

## Introduction

Pollutant levels in the environment must be closely monitored to ensure the safety of both people and the planet. Everyone knows that metal ions are crucial to many biological and chemical reactions. Undoubtedly, advances in the invention of luminescent probes for detecting metal ions have greatly aided knowledge of how to determine metal ions in environmental and biological samples. There are various applications for aluminium because in the earth's crust, aluminum is the third most frequent element and the most abundant metal [1]. Several diseases are linked to aluminum ion overload in the human body, including microcytic hypochromic anaemia, encephalopathy, bone softening, Alzheimer's disease, and myopathy [2].

However, aluminium may not always have beneficial effects, even although it is a plentiful part of soils and rocks. For instance, when silicate minerals are exposed to low-pH liquids such as acid rain, it dissolves from them, increasing the amount of free aluminum ions in the environment, which is harmful to organisms. Additionally, the existence of soluble aluminium ions is among the major causes of acidic soil, and aluminium toxicity is responsible for about fourty percent of the acid soils of the world and cause root stunting in plants [2, 3]. Additionally, long-term exposure or consumption to aluminium ions can also lead to illness in humans through the accumulation of aluminium ions in numerous organs [4–9]. Significantly when there are a lot of aluminum ions in brain tissue, they damage the central nervous system badly and make neurologic diseases more possible [9]. The World Health Organization (WHO) sets the upper limit for aluminum ions in drinking water at 7.4 µM. As a result, it is crucial to be able to identify extremely low concentrations of aluminum ion in real samples. There are a number of quantitative and qualitative techniques for detecting aluminium ions [10], like Atomic Absorption Spectrometry [11] and ICP atomic emission spectrometry [12], which are mostly time-consuming and expensive. Fluorescence, on the other hand, stands out because to its superior sensitivity, high temporal resolution, and portability [13–18]. Several luminescent alumium probes were reported [19–22]. However, in aqueous solutions, the ion's high hydration generally results in a poor coordination ability that is easily interfered with by other chemicals. Consequently, scientists are still working to develop a practical, and sensitive luminescence probe for detection aluminum ion in real samples. Unfortunately, most currently reported luminescent probes need time-consuming and laborious synthetic methods before they can be used in actual samples. In comparison to luminescent probes with a turn-on mode, ratiometric probes can efficiently reduce the majority of external interferences due to the rectification of two emission bands. Consequently, ratiometric-mode sensing offers significant potential for use with complicated samples [23–26].

The principle of fluorescence energy transfer is an effective way for designing ratiometric luminescence probes since the probes emit at two distinct wavelengths under single wavelength of excitation. The fluorescence energy transfer is a mechanism in which the excitation energy is absorbed by a donor group then transferred it to an acceptor group of the system. Donor–acceptor distance and spectral overlap play major roles in determining the efficiency of fluorescence energy transfer [27, 28]. This process can provide an efficient way to eliminate self-quenching and fluorescence detection error of the probe [29]. In particular, very few probes which based on this process for aluminum ion detection were reported [30–32]. Even while these probes have shown promising properties—including high selectivity and sensitivity—they still have a few drawbacks, most of which are rooted in the use of rhodamine. Therefore, it is preferable to design new ratiometric probes based on energy transfer process for aluminum ions with enhanced detection limits in environmental samples. So, for effective fluorescence energy transfer process, it is necessary that the emission spectra of the donor overlap with the absorption spectra of the acceptor, making the selection of acceptable “Donor–Acceptor” couples a significant task [33]. Furthermore, visual analyte monitoring is convenient and advantageous for both quick qualitative and semiquantitative evaluation [34]. So, the visual assay is crucial for regular analysis and real-time monitoring of environmental contaminants. Few visual approaches for detecting aluminum ions in the presence of other elements were reported so far [35].

In recent years, researchers have investigated the use of lanthanide complexes (particularly Eu(III) and Tb(III)), to create selective and sensitive detection techniques for environmental analyte [36, 37]. The major benefits of this technique are narrow emission bands, long liftimes, longer emission wavelength, and significant Stokes shifts. Thier properties could be utlized to reduce background autofluorescence and scattering interference from the sample matrix. Luminescent lanthanide complexes based on β-diketones may give excellent luminescent responses with visible colours emission, implying a possibility for these complexes to be utilized into attractive chemical sensors [38, 39]. The β-diketone ligands are one of the crucial “antennas” because of their ability to transfer energy to metal ions [33, 40, 41].

In this work, the complex of Eu(III) with 3-(2-naphthoyl)-1,1,1,-trifluoro acetone (3-NTA) in methanol was chosen as a ratiometric luminescent sensor for detecting aluminum ions in water samples. In previous studies, ratiometric luminescent probes for determining aluminum ions were based on organic compounds [35, 42] and carbon dots [43, 44]. Our probe is distinct from those mentioned above since it incorporates lanthanide metal ions (Eu^3+^) which have luminescence color changed from red to bright green and dark green. Also, our probe was characterized by a high stock shift compared to others. According to our knewlodge, this is the first ratiometric probe based on luminescence lanthanide complex to detect aluminum ions. The approach is rely on the emission change of the europium complex after adding different concentrations of aluminum ions. The luminescence of the probe is monitored at 615 nm (for Eu(III) and the emission wavelength of the ligand at 480 nm using λec= 333 nm. By adding aluminum ions, the emission of the probe at 615 nm decreased, and the emission at 480 nm increased. The sensing of aluminum ions was evaluated by graphing the luminescence intensity ratio at 480 nm to 615 nm vers aluminum ion concentrations. In comparison to other examined metal ions, the probe displayed strong selectivity for aluminum ions. Additionally, the concentration of aluminum ion can be estimated semi-quantitatively by visually observing the luminescence colour change of the probe from red to light green and then to dark green upon excitation after being excitated by a UV lamp with 365 nm. The proposed probe has been successfully applied to determining aluminum ions in acutal water samples with significant results.

## Experimental

### Chemicals

Eu(NO_3_)_3_.6H_2_O and 3-(2-naphthoyl)-1,1,1,-trifluoro acetone, 99% (3-NTA) were bought from Sigma–Aldrich (www.sigmaaldrich.com). All inorganic salts used were also bought from Sigma–Aldrich (www.sigmaaldrich.com). All solvents were of analytical grade and utilized without any purification.

### Instrumentation

The UV-VIS spectra were measured using a Shimadzu UV-1800 Spectrophotometer. (https://www.shimadzu.com) using a 1.0 cm of path length quartz cell. The luminescence spectra were obtained using a Jasco 6300 spectrofluorometer (https://jascoinc.com) using a 1.0 cm of path length quartz cell and a 150 W xenon lamp for excitation. The bandwidths of emission and excitation were 5 nm. The infrared spectra were obtained in the 4000–500cm^−1^ region using Bruker FTIR Alpha Spectrometer (https://www.bruker.com).

### Solutions preparation

1.0 x 10^−3^ M stock solutions of metal ions (Mn^2+^, Fe^2+^, Cu^2+^, Al^3+^, Zn^2+^, Ni^2+^, Cd^2+^ and Pb^2+^) were prepared in deionized water. 1.0 x 10^−3^ M of Eu(NO_3_)_3_.6 H_2_O stock solution was prepared by dissolving 0.00446 g in 10.00 mL of methanol. The 1.0 x 10^−3^ of M 3-NTA stock solution was prepared through dissolving 0.00266 g in 10.00 mL methanol. To study the sensitivity and selectivity of our probe towards aluminum ions, the working solutions were freshly prepared by mixing various volumes of Al(III) stock solutions (0, 10, 20, 30, 50, 80, 100, 150, 200, 250, 300, 350, 400, 450, 500 and 1000 µL) with a fixed volume (100 µL) of 1.0 x10^−3^ M Eu(III) stock solution and (300 µl) of 1.0 x10^−3^ M 3-NTA stock solution in a 10 mL volumetric flask then completing the solution with methanol. This gives Al^3+^ concentrations of 0, 1, 2, 3, 5, 8, 10, 15, 20, 25, 30, 35, 40, 45, 50, and 100 µM, respectively. Finally, the spectroscopic measurements were performed.

### Luminescence determination of Al(III)

The emission spectra of the Eu(III)-(3NTA)_3_ complex were recorded and monitored at two emission wavelengths (λ_em_ = 480 and 615 nm) in methanol using λ_ex_= 333 nm. The analysis was done via monitoring the luminescence quenching at 615 nm and the luminescence enhanced at 480 nm of the complex in the existance and absence of various concentrations of Al(III). The luminescence emissions were monitored after being incubated for fives minutes at room temperature. The measurements were carried out three times, and the average luminescence intensities were computed. The quantification of aluminum ions using ratiometric method was done by plotting the luminescence ratio (F_480nm_/F_615nm_) *versus* the concentration of aluminum ions.

### Water samples preparation

Tap water was collected after 10 min of flowing from taps prescent in a lab inside our university. Bottled mineral water samples were bought from a local market (Dasani company). These samples were tested without any pretreatment. Sea water samples were collected from Port Said city, Egypt. Bottles of 1000 milliliters were used to collect the water samples. We used Whatman No. 41 filter paper to filter the water samples, then transferred 100 mL of each filtered sample to a 250 mL conical flask and added 10 mL of a mixture of HNO_3_ and H_2_O_2_ (1:9, v/v). This sample set was heated in a reflux for 1.5 hours to oxidize organic matter. After being cooled, the samples were moved to a 100 mL volumetric flask, where they were brought up to the correct volume with deionized distilled water, well mixed, and analyzed using the described procedure. The standard addition method detected Al(III) in water samples. All spectroscopic measurements were performed at a constant temperature of 25 ± 1 °C (room temperature). We performed the measurement three times and the average value was calculated.

## Results and discussion

### Spectral properties of the probe

**Fig. 1:**
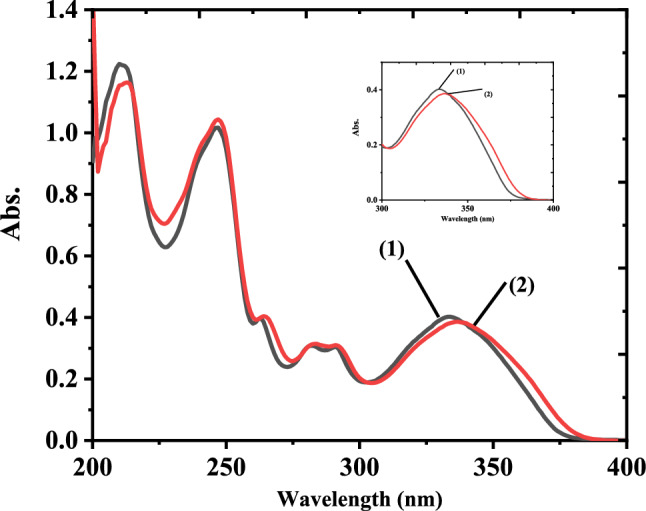
Absorption spectra of (1) 3 × 10^–5^ M 3-NTA and (2) 1 × 10^–5^ M Eu(III) + 3 × 10^–5^ M (3-NTA)_3_ in methanol at room temperature

The absorption spectra of the 3-NTA and its complex with europium ion were measured in methanol in the range of 200–400 nm at room temperature, as shown in Fig.1. The absorption band for 3-NTA is at λ_max_ = 333 nm, due to the π-π* enol absorption of the ligand (3-NTA) [45] (ε_333nm_ = 1.37 x 10^4^ M^−1^ cm^−1^). This band was shifted to 337 nm upon addition of Eu(III) (red shift). This bathocromic shifte attributed to the binding between europium ion and 3-NTA. The molar absorbitivity of the complex at 337 nm was calculated to be (3.95 x 10^5^ M^−1^ cm^−1^). After testing the complex with a UV-Vis spectrophotometer for a week, we found that it was stable.

**Fig. 2:**
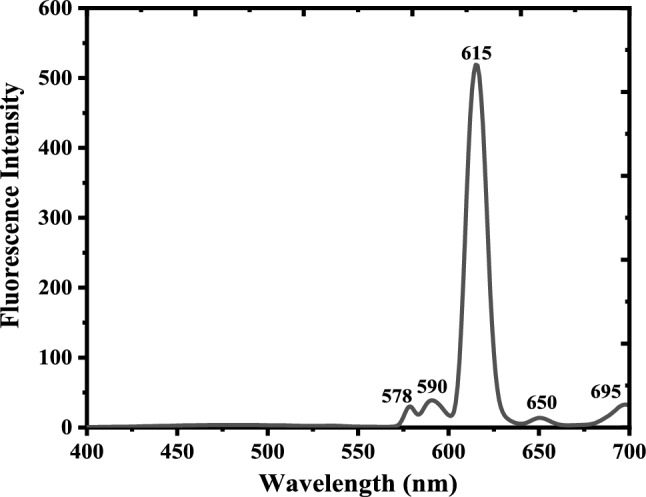
Luminescence spectra of 1 × 10^–5^ M of Eu(III)-(3-NTA)_3_ complex in methanol at room temperature. The excitation wavelength was 333 nm and emission and excitation slit widths were 5 nm, respectively

The luminescence spectra of the probe revealed five distinct emission bands characteristic of the eurpium ion at 675, 650, 615, 590, and 578 nm, which are correspond to the ^5^D_0_ -> ^7^F_4_ , ^5^D_0_ -> ^7^F_3_ , ^5^D_0_ -> ^7^F_2 ,_^5^D_0_ -> ^7^F_1_, and ^5^D_0_ -> ^7^F_0_, transitions, respectively, as shown in Fig. 2. By comparison to other emission bands, the intensity of the emission at 615 nm was the strongest which is responsible to make the complex emits an obvious red color. The emission intensity of the Eu(III)-(3-NTA)_3_ was obviously high, indicating that the Förster resonance energy transfer (FRET) which take place through absorption excitation energy by 3-NTA then transferred to Eu(III) is sufficiently efficient. This can be attributed to the great degree of overlap between the 3-NTA emission (donor) and absorption spectra of Eu(III). Also, the comparatively small gap between the T_1_ state of 3-NTA and the Eu(III) resonance level might explain the efficient energy transfer.

**Fig. 3:**
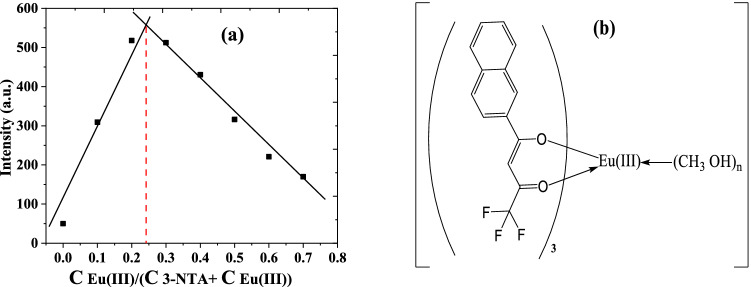
**a** Job plot of Eu(III)-(3-NTA) system at λ_ex/em_ = 333/615 nm in methanol at room temperature [Eu(III)] = [3-NTA] = 1 × 10^–4^ M **b** The hypothesized structure of the Eu (III)–(3-NTA)_3_ complex in methanol

The stoichiometry of the complex produced between europium ion and 3-NTA was determined using a Job plot, as shown in Fig. 3a. The figure clearly shows that the maximum luminescence intensity occurs at a mole fraction of 0.25 of europium ion. This finding demonstrates that one europium ion forms a complex with three molecules of 3-NTA, which is denoted as Eu(III)-(3-NTA)_3_. According to the antenna theory [46], by raising the concentration of 3-NTA until the molar ratio [3-NTA]/[Eu(III)] = 3:1, the emission of Eu(III) is enhanced, implying the creation of a Eu(III)-(3-NTA)_3_ complex (Figure 3b). When more 3-NTA is added, the emission intensity of europium ion is reduced because the extra ligand molecules cause self-quenching.

### Response of the probe to Al^3+^

**Scheme 1:**
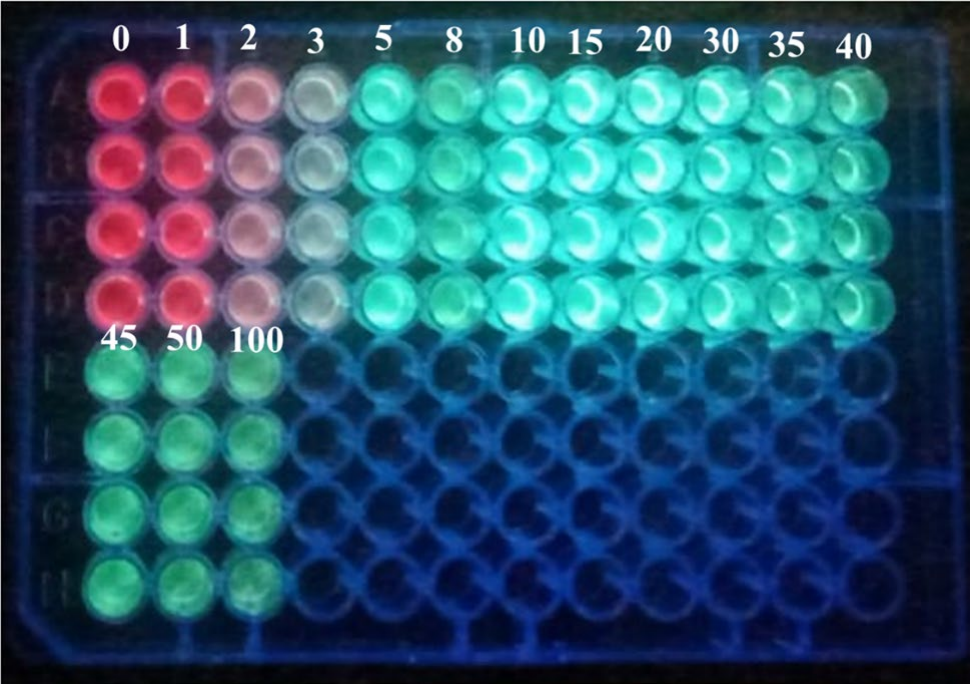
Schematic illustration of visual quantitation of Al(III) in a microtiter plate with the Eu(III)-(3-NTA)3 complex as a luminescent probe. The probe was reacted with different concentrations of Al(III) and excited under UV light with* λ*_exc_ = 365 nm (the numbers indicate the concentrations of Al(III) in µM)

The luminescence spectra of Eu(III)-(3-NTA)_3_ (10 μM) were recorded in methanol. When the probe excited at 333 nm, it exhibited an emission band specialist of the Eu(III) ion at 615 nm. By adding Al^3+^, the emission of the probe at 615 nm (for Eu(III)) decreased, and the emission of the ligand at 480 nm increased at the same time, leading in the suppression of the luminescence probe's FRET process (Fig.ure 4). Thus, the complex showed a ratiometric luminescent response upon adding an aluminum ion which restricted the process of FRET that happen, and the luminescent color of the probe changed from red to green under UV light (365 nm, Scheme 1). At optimum conditions, our probe's calibration curve for measuring Al^3+^ concentrations is expressed through a relationship between the luminescence intensity ratio (F_480_/F_615_) vs. the Al^3+^ concentrations (Figure 5). The calibration plot has a sigmoidal shape. The calibration reveals a dynamic range from 0.1 to 100 μM (Fig.ure 5, Table 1) obtained by a sigmoidal fit (R^2^=1, *n*=3). The calibration plot has a linear range (Fig.ure 5 (inside)) between 5.0 and 45 μM. It can be described by F_480_/F_615_= 0.22 (±0.0027)⋅c(Al^3+^) (μM)–1.03 (±0.0196) (R^2^=0.9989, *n*=3). F480/F615 ≠ 0 at c(Al^3+^) = 0 due to the prescence of very small emission intensity of the 3-NTA. To take advantage of the full dynamic range, a sigmoidal fit from 0.1 to 100 μM yielded a perfect coefficient of determination (R^2^=1, *n*=3). The respective fitting parameters are shown in Table 1. The detection limit (3σ/slope) of the probe was 0.27 µM (7.75 × 10^−3^ mg/L), which well meets the acceptable limit for Al^3+^ in drinking water. The LOQ is 0.89 µM. The calibration curve for aluminum ion in methanol was made by repeating at least fourteen concentrations three times. It shows that the probe is appropriate for measuring Al(III) in water samples. Table 2 lists the several chemosensors used to determine Al (III) [35, 47–54]. When we compared earlier approaches to our suggested method, we noticed that our method is more sensitive than others. Moreover, the limit of detection obtained by our method is more adequate for investigating Al(III) concentrations in the environmental samples. Our proposed approach offers a broader linear range. As a consequence, most samples will not need further dilution. The proposed method is more appropriate for environmental samples. Where methanol used for strengthening the luminescence of the probe is harmful for living things. In vivo Al^3+^ detection is impossible but the detection of Al^3+^ can be determined in vitro instead of vivo. The real samples in case of blood plasma, show background fluorescence from their considerable content of proteins and other intrinsically luminescent compounds. The presence of this background fluorescence in blood plasma would certainly interfere and overlap with the emission of Eu(III)-(3-NTA)_3_ in the 350–500 nm emission range. This would considerably decrease the sensitivity of determination of Al^3+^. Methanol solvent can be useful to avoid such interference and to reduce scatter and autofluorescence from real samples, through precipitating the protein then removing it by centrifuge during extraction of Al^3+^ from blood samples by methanol.

**Fig. 4:**
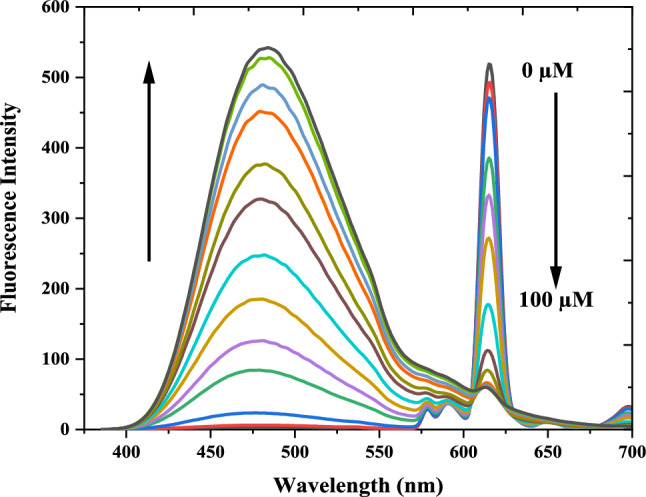
Luminescence emission spectra of a 10.0 μM Eu(III)-(3-NTA)_3_ in presence of Al(III) at 0, 1, 2, 3, 5, 8, 10, 15, 20, 25, 30, 35, 40, 45, 50 and 100 μM (from top to bottom; λ_exc_ = 333.0 nm; methanol)

**Fig. 5:**
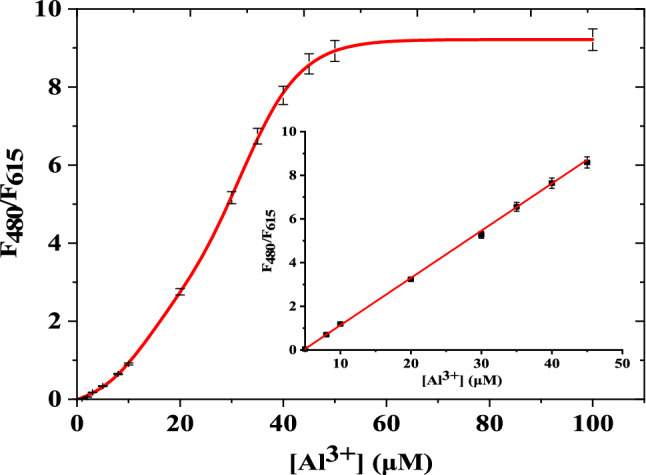
The luminesce intensity ratio of Eu(III)-(3-NTA)_3_ (10 μM, methanol) as a function of Al(III) concentration. λ_ex_ = 333 nm, in methanol at room temperature

**Table 1:** Fitting parameters for sigmoidal fits of the calibration plot (*n* = 3)

Model	Luminescence evaluation
Equation	$y=A1+\left(A2-A1\right)[\frac{p}{1+{10}^{\left(LOGx01-x\right)h1}}+ \frac{1-p}{1+{10}^{\left(LOGx02-x\right)h2}}]$
A1	− 0.237 ± 0.161
A2	9.214 ± 0.041
LOGx01	12.666 ± 2.374
LOGx02	32.025 ± 1.186
h1	0.087 ± 0.029
h2	0.075 ± 0.005
*p*	0.291 ± 0.107
*R* Square (COD)	1.000
Adj. *R* Square	1.000

**Table 2:** Comparison of Eu(III)-(3-NTA)_3_ probe and others probe for the determination of Al^3+^

The Probe	Detection metal ions	Detection method	Recognition mechanism	Detection solvent	linear range (LOD)	Applications	References
Eu(III)-(3-NTA)_3_	Al^3+^	Ratiometric luminescence Stoke’s shift (~ 131 nm)	FRET	methanol	0.27–100 µM (0.27 µM)	Tap, mineral, and sea water samples	Our work
A coumarin-quinoline based probe	Al^3+^	ratiometric fluorescence Stoke’s shift (~ 90 nm)	FRET	Ethanol/water	0.11–1.25 µM (0.24 µM)	lake water samples	[34]
RS5 compound	Al^3+^, Fe^2+^, Fe^3+^	Ratiometric fluorescence Stoke’s shift (~ 45 nm)	Intramolecular charge transfer (ICT) transition along with chelation enhanced fluorescence (CHEF) processes	H_2_O-DMSO (1:1, v/v) medium	0.30 – 1 µM (0.30 µM)	deep well water, tap water, drinking water, pond water, river water, bovine serum albumin (BSA) solution and blood serum	[47]
Rhodamine-derived Schiff base	Al^3+^, Cu^2+^, Fe^3+^	Colorimetric response to Cu^2+^ and Al^3+^, and “off–on” fluorescence response toward Fe^3+^	PET	semi-aqueous media	0.011–10 µM (11 nM)	NA	[48]
7-hydroxy-6-[(2-hydroxy-naphthalen-1-ylmethylene)-amino]-4-methyl-chroman-2-one	Al^3+^	“on–off” fluorescence response Single wavelength	ESIPT	H_2_O	0.1–1 µM (0.1 µM)	living HeLa cells. and in vivo zebrafish	[49]
(E)-*N'*-(3-(benzo[d]thiazol-2-yl)-2-hydroxy-5-methylbenzylidene)-7-(diethylamino)-2-oxo-2*H*-chromene-3-carbohydrazide (CHS)	Al^3+^ and Pyrophosphate	“off–on” fluorescence response Single wavelength	NA	DMSO/HEPES (4:1 v/v, pH = 7.4) buffer system	0.16–0.5 µM (0.16 µM)	living HeLa cells	[50]
(E)-1-(((2-(phenylthio)phenyl)imino)methyl)naphthalen-2-ol (NAPTA)	Al^3+^	chromogenic and turn-on fluorescence behavior	CHEF and ICT	DMSO	(1.3 µM)	NA	[51]
(E)–2-(benzo[d]thiazol–2-yl)–4-(4-(diethylamino)–2-hydroxybenzylideneamino)phenol (HBTA)	Al^3+^	“off–on” fluorescence response Single wavelength	Aggregation induced emission	THF: water(9:1 v/v)	(0.87 µM)	NA	[52]
2-hydroxy-1-naphthaldehyde-2-amino thiazole	Al^3+^	“off–on” fluorescence response Single wavelength	CHEF	DMSO:H_2_O (1:1 v/v)	(1.3 µM)	Breast Carcinoma in Human	[53]
Probe NA	Al^3+^, Zn^2+^	“off–on” fluorescence response Single wavelength	CHEF	CH_3_CN:H_2_O (7:3, v/v)	(74.0 μM)	lakes, groundwater, and tap water	[54]

*NA* not available

For a visual readout, Scheme 1 displays a photograph of a sensing microtiter plate illustrating the variation of color that arises after the interaction between the complex with various concentrations of Al^3+^ during exposur to UV lamp with 365 nm. In the absence of aluminum ions, the probe is deep red, but when the concentration of aluminum ion is increased, the color shifts to a lighter red that fades away gradually after 1-3 mM of aluminum ion have been added. Then, a light green color appears as a transition at 5–30 µM and changes into a deep green at higher aluminum ion concentrations. This is in agreement with the emission spectra depicted in Fig. 4, which demonstrates that when aluminum ion concentrations increase, greener light is released while red europium emission decreases. Hence, a UV lamp can be used as instrumentation for a visual and semiquantitative assessment of aluminum ion in three concentration ranges.

**Fig. 6:**
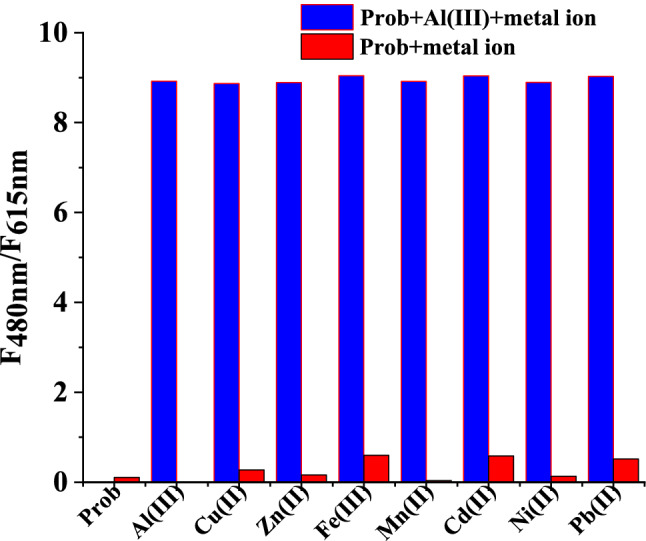
Ratio luminescence intensity (F_480nm_/F_615nm_) of the probe (10 μM). Red bars: ratio luminescence intensity of the probe with the addition of the other metal ions (10 eq.). Blue bars: ration luminescence intensity of the probe with the addition of the other competing ions (10 eq.) and Al^3+^ (10 eq.). in methanol at λ_ex_ = 333 nm, scan range 400–700 nm, slit width 5 nm

The luminesce behavior of Eu(III)-(3-NTA)_3_ complex in the prescence of other metal ions (Cd^2+^, Pb^2+^, Zn^2+^, Ni^2+^, Cu^2+^, Co^2+^, Mn^2+^, and Fe^3+^) was studied (see Fig. S1). It was noticed that, the emmsion of the probe at 615 nm decreased while the emission at 480 nm was not appeared in the presence of different concentrations of metal ions under study. Only the Al^3+^ cause a significant change of the (F_484 nm_/ F_615 nm_) value under comparable conditions through the restriction of the FERT process between 3-NTA and Eu(III) ion. However, in the case of other metal ions, they quenched the emission of the prob at the characteristic luminescent band of europium ion at 615 nm without restricting the FERT process between 3-NTA and Eu(III) ion. They just quenched the emission band of the probe at 615 nm without the appearance of the emission band of 3-NTA, as did in the case of Al(III). Moreover, as shown in Fig. 6, the addition of metal ions under study to the probe in the prescence of aluminum ions did not show a significant change on the (F_480 nm_/ F_615 nm_) value of the probe. All of these data suggested that the probe's selectivity for aluminum ions over other competing metal ions was high.

### Effect of response time

For investigation of the best response time of our probe, the (F_480 nm_/ F_615 nm_) value was recorded at a time interval of five min for one hour, and the results are displayed in Fig.ure 7. After five minutes, more than 90 percent of the total signal change has occurred for the majority of concentrations (within 1.0 hours). Due to our desire to develop a rapid Al(III) sensing technique, we have fixed the measurement response time at five minutes.

**Fig. 7:**
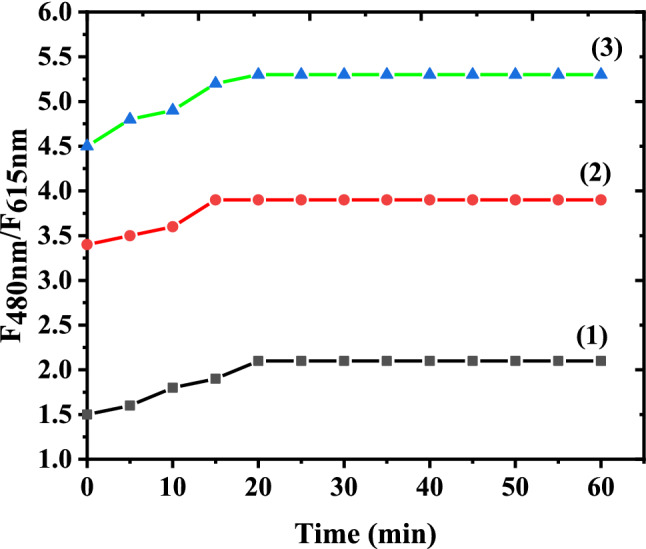
Ratio luminescence intensity (F_480nm_/F_615nm_) of the prob (10 μM) in presence of a Al(III) with different concentrations at different times. The concentrations of Al(III) (μM) are: (1) 10.0, (2) 20.0 and (3) 30.0, respectively

### The proposed mechanism

The proposed mechanism for the interaction between the Eu(III)-(3-NTA)_3_ probe and the Al(III) is depicted in Scheme 1. The following is an explanation of the suggested mechansium: The europium ion is widely recognized for its distinctive red emission at 591 and 615 nm, due to to the ^5^D_0_ -^7^F_1_ and ^5^D_0_ -^7^F_2_ transitions, respectively. Emission intensity of the Eu(III) through direct excitation is relatively weak. To overcome this, organic ligand was utilized to synthesize Eu(III) complex in which higher emission intensity can be observed via the antenna effect [46]. It is feasible to use sensitive excitation of eurpouim ion by an appropriate organic ligand through energy transfer [55]. According to Förster's resonance energy transfer theory (FRET), the rate of energy transfer is determined by the degree of overlap of the donor's emission spectra with the accepter's excitation spectra and the distance between them [56]. An organic ligand with a high triplet state population considered an efficient sensitizer where, the energy transfer happens from the triplet state of the ligand to excited stat of the lanthanide ions. Additionally, it is known that, the luminescent lanthanide complexes based on β-diketones may produce bright visible-emitting signals, indicating great potential for these complexes as good chemical sensors [38, 39]. 3-(2-naphthoyl)-1,1,1,-trifluoro acetone (3-NTA) is one of the β-diketones that display an effective energy tranfer from its T1 state to the emssive levels of europium ions. So, the Eu(III)-(3-NTA)_3_ complex would show a sensitized luminescence band at 615 nm through the Förster’s resonance energy transfer, where 3-NTA acts as an energy donor, and the europium ion acts as an energy acceptor, after excitation of the 3-NTA moiety at λ_ex_ = 333 nm,. Upon adding Al^3+^, a new emission band at 480 nm appeared. Furthermore, the main peak of the europium ion at 615 nm was fixed and quenched with increasing concentrations of aluminum ions. The data indicate the binding of aluminum ions with 3-NTA. This binding restricted the FRET process between 3-NTA and Eu(III). The data also confirm the formation of a stable complex between 3-NTA and Al^3+^. This complex exhibits relatively high fluorescence at 480 nm as a result of the intensified intramolecular charge transfer process (ICT) and the chelation enhanced fluorescence (CHEF) effect, both of which are evidenced by the red-shifted emission maxima of 3-NTA upon interaction with Al(III) [57].

**Scheme 2:**
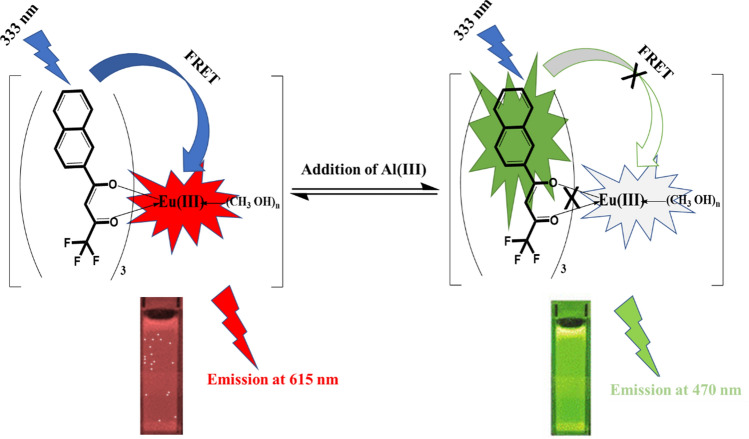
Schematic representation the interaction between the Eu(III)-(3-NTA)3 probe and Al(III) based on FRET process.

For more confirmation, the UV–vis absorption spectra of the Eu(III)-(3-NTA)_3_ were recorded in methanol medium in the absence and existance of aluminium ion as shown in Fig. S2. The probe showed absorption bands at 213 and 246 nm due to π–π* transitions and a broad band at 337 nm due to ICT. The addition of aluminum ion to the probe had no discernible effect on its UV–vis spectrum, reviling that the probe binds to aluminum ion in the excited state [58].

The IR spectra of 3-HNTA, Eu(III)-(3-NTA)_3_ probe and Eu(III)-(3-NTA)_3_ with Al(III) were measured in methanol (Fig. S3). The figure displays a similar infrared absorption bands for the three systems. The band at 3323 cm^−1^ confirms the presence of hydroxyl group. Furthermore, the absorption bands at 2831 and 1456 cm^−1^ are due to methylene groups of 3-NTA. Nevertheless, Figure 3b shows a prominent band at 1666 cm^−1^ due to C=O group. Upon addition of one equv. of alumium ion to the complex the peak at 1666 cm^−1^ disapered. This confirm the displacement of Eu(III) ion in the probe by Al(III) ion.

### Analytical application

To examine the practical use of the probe for the detection of Al(III) in tap water, mineral water, and salt water, a standard addition experiment was utilized [35, 59]. Sample solutions with known concentrations of Al(III) were used to evaluate the probe's utility, as indicated in Table 3. It was noticed that, both the added and detected aluminum ions concentrations utilizing the probe showed promising findings. The measurements were conducted three times. All the values of recovery were between 94.50 and 107.50 % with RSD% range between 2.6–7.89, confirming the reliability of the probe (Eu(III)-(3-NTA)_3_) for determing alumnium ions in real water samples.

**Table 3:** Recovery study of Al^3+^ by Eu(III)-(3-NTA)_3_ probe in water samples

Sample	[Al^3+^] (μM) added	[Al^3+^] (μM) found mean^a^ ± SD	Recovery rate (%)	RSD (% *n* = 3)
Mineral water	10	10.30 ± 0.1	103.0	5.23
	20	19.28 ± 0.3	96.25	2.60
	30	29.53 ± 0.2	98.42	3.74
Tap water	10	9.94 ± 0.08	99.44	6.45
	20	21.35 ± 0.3	106.75	5.69
	30	29.51 ± 0.5	98.37	4.56
Sea water 1	10	10.53 ± 0.4	105.30	2.14
	20	21.50 ± 0.2	107.50	7.56
	30	28.69 ± 0.6	95.65	6.23
Sea water 2	10	10.27 ± 0.1	102.70	4.25
	20	18.90 ± 0.7	94.50	6.26
	30	28.99 ± 0.3	96.63	7.89

*SD* Standard Deviation, *RSD* Relative Standard Deviation

^a^Mean for three determination

## Conclusions

In summary, we presented a new ratiometric luminescent probe of Eu(III)-(3-NTA)_3,_ which is fast-responding and possesses good selectivity and sensitivity towards aluminum ions with a detection limit of 0.27 μM. The method relies on the luminescence change of the Eu(III) complex with 3-(2-naphthoyl)-1,1,1,-trifluoro acetone (3-NTA) after interaction with various concentration of aluminum ions. The luminescence of the probe is monitored at the characteristic emission wavelength of europuim ion at 615 nm and the emission wavelength of the ligand at 470 nm under excitation at 333 nm. The addition of aluminum ion suppressed the Eu(III) emission at 615 nm under 333 nm excitation, while simultaneously enhancing the ligand emission at 470 nm. Optimum detection was obtained in methanol. The quantification of aluminum ion using ratiometric method was determined by plotting the luminescence ratio (F_470nm_/F_615nm_) *versus* Al(III) ion concentration. We have also shown that the Eu(III)-(3-NTA)_3_ may be used to measure the concentration of aluminum ion in actual samples, like tap, mineral, and seawater

## Supplementary Information

Below is the link to the electronic supplementary material.

Supplementary file1 (DOCX 735 KB)

## Data Availability

All data generated or analyzed during this study are included in this published article [and its supplementary information files].
