# Supplementary material for: Sensitive ratiometric sensor for Al(III) detection in water samples using luminescence or eye-vision
Source: Anal Sci. 2023 Apr 18;39(8):1307–16. doi: 10.1007/s44211-023-00340-6 (PMC10359221; doi:10.1007/s44211-023-00340-6)
Supplement: Supplementary file 1 — Supplementary file1 (DOCX 735 KB) [file 44211_2023_340_MOESM1_ESM.docx]

**Supporting Information**

|  |  |
| --- | --- |
|  |  |
|  |  |
| Figure S1 The luminesce spectra of 10 µM of Eu(III)-(3-NTA)_3_ complex in the presence of different metal in methanol at room temperature (λ_ex_ = 333nm). | |

Figure (S2). Absorption spectra of 1 x 10^-5^ M Eu(III)+ 3 x10^-5^ M (3-NTA)_3_ in the absence and presence of 1x10^-5^ M A(III) in methanol at room temperature

|  |  |
| --- | --- |
| Fig. S3. IR spectrum of 3x10^-5^ M 3-NTA (Black color), 1 x10^-5^M Eu(III)-(3-NTA)_3_ complex (Red Color) and 1 x10^-5^ M Eu(III)-(3-NTA)_3_ complex + 1x10-5M Al(III) (Blue color) in methanol. | |
